# Different Optical Behaviors Revealed by Electroluminescence and Photoluminescence of InGaN/GaN Coupled Quantum Wells

**DOI:** 10.3390/nano14181523

**Published:** 2024-09-20

**Authors:** Huan Xu, Yachao Wang, Xin Hou, Wei Ou, Tao Yang, Yang Mei, Baoping Zhang

**Affiliations:** 1 Laboratory of Micro/Nano-Optoelectronics, Department of Microelectronics and Integrated Circuits, Xiamen University, Xiamen 361005, China; xuhuan@stu.xmu.edu.cn (H.X.); 36120210156001@stu.xmu.edu.cn (Y.W.); houxin@stu.xmu.edu.cn (X.H.); 33320210155987@stu.xmu.edu.cn (T.Y.); 2 Institute of Nanoscience and Applications, Southern University of Science and Technology, Shenzhen 518055, China; 36120200155820@stu.xmu.edu.cn

**Keywords:** coupled quantum well, photoluminescence, electroluminescence

## Abstract

The optical properties of wurtzite violet InGaN/GaN coupled quantum well (QW) structures are experimentally studied using photoluminescence (PL) and electroluminescence (EL) spectroscopy. Two emission peaks, referred to as Peak H and Peak L, are observed in both PL and EL spectra, due to the ground state splitting induced by the well coupling. Experimental PL and EL results reveal that coupled QWs show different optical responses due to the different variation in the electric field inside the QW structure. Since the direction of the polarization electric field of the as-grown well/barrier layers is different, the external electric field applied by electrodes can change the energy band alignment between the well and the barrier layers, thus adjusting the coupling between the wells. Our results provide relevant information to improve our understanding of the optical properties of InGaN/GaN QWs and to develop novel optoelectronic devices.

## 1. Introduction

Gallium nitride (GaN)-based optoelectronic devices, such as light-emitting diodes (LEDs) and laser diodes, are widely used in lighting, display, and biomedicine. However, due to the low hole mobility, optoelectronic devices based on conventional InGaN/GaN multiple quantum well structures suffer from carrier transport problems [1]. The well far from the p-GaN may become an absorption well due to insufficient hole injection, resulting in a reduction in the output power. In coupled quantum well (CQW) structures, narrower quantum barriers are used to separate the QWs [2], which improves the inter-well hole transport by the carrier tunneling effect. This enhances the homogeneity of the carrier distribution compared to conventional QW structures, which is helpful for improving the laser power and reducing the threshold current [3]. The fundamental physical properties in GaAs-based CQWs have been studied in detail and exploited for manufacturing optoelectronics devices [4,5,6,7,8,9].

Compared with GaAs, nitrides with wurtzite crystal structures do not possess a central symmetry and thus show a spontaneous polarization effect [10]. At the same time, due to the difference in lattice constants between GaN and InN, piezoelectric polarization is present in InGaN/GaN QWs. The net electric field in the well leads to the well-known quantum confined Stark effect (QCSE), which is detrimental to the optoelectronic performance of devices [11]. In contrast, the energy band GaAs-based QWs are flat [12] because there are no spontaneous and piezoelectric polarizations. The GaN barrier is subjected to tensile stress, and the direction of piezoelectric polarization is the same as that of spontaneous polarization. However, the direction of piezoelectric polarization in the InGaN well layer is instead opposite to the spontaneous polarization because of compressive stress. Overall, the energy bands of the InGaN well layer and GaN barrier are bent in opposite directions, which will definitely affect the coupling behavior between adjacent wells.

**Figure 1:**
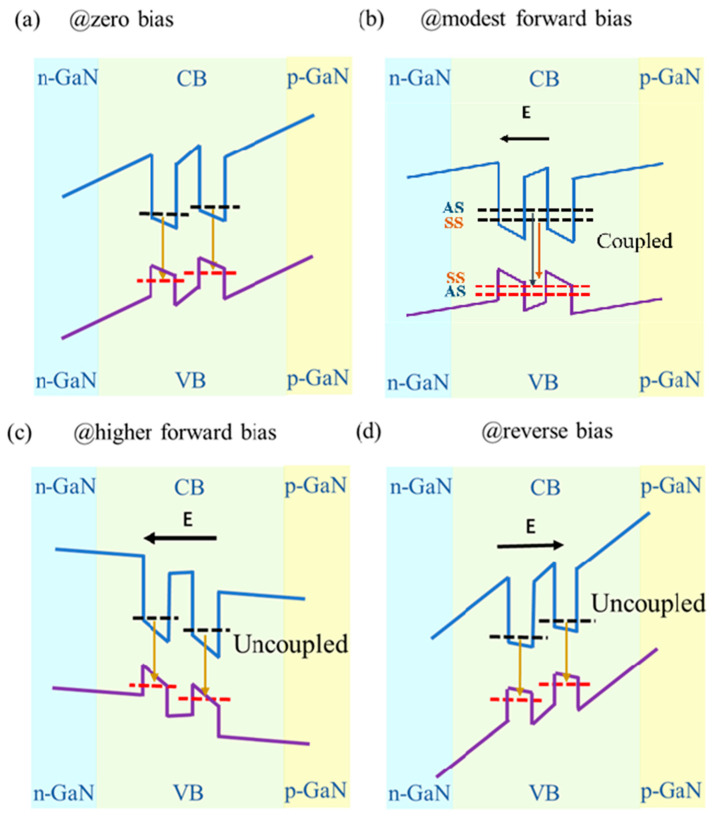
The band structure of coupled InGaN/GaN QWs for (**a**) zero bias (ZB), (**b**) modest forward bias (FB) with coupling, (**c**) higher forward bias with uncoupling, and (**d**) reverse bias (RB) with uncoupling in EL. The acronyms SS and AS refer to symmetric and antisymmetric extended states in the CQW structure, respectively.

Figure 1 shows the band structure of coupled InGaN/GaN QWs for (a) zero bias (ZB), (b) modest forward bias (FB) with coupling, (c) higher forward bias with uncoupling, and (d) reverse bias (RB) with uncoupling in EL. The band structure of coupled InGaN/GaN QWs is a predicted one but is in good agreement with the experimental results. The acronyms SS and AS refer to symmetric and antisymmetric extended states, respectively. E denotes the applied electric field, whereas CB and VB denote conduction and valence band, respectively. The applied electric field also influences the energy levels of QWs when CQWs are used to build optoelectronic devices. It has been reported that an external electric field is required to effectively shield the internal field of the p-n junction in GaAs-based CQW devices [13]. In the case of InGaN/GaN CQW devices, the situation is more complicated. In fact, the electric field on the energy band of the well and barrier layers has opposite effects due to the different directions of the net polarization between the well and barrier layers. At zero bias, due to the influence of polarization, the electron and hole energy levels of adjacent QWs are subject to Stark shift and are different [14], i.e., they are uncoupled; see Figure 1a. When a forward bias electric field is applied, the polarization in the InGaN well is oriented in the same direction as the applied electric field, and the bending of the energy bands is enhanced, while that of the GaN barrier is weakened. Considering the QCSE, the energy bands of the InGaN well layer and GaN barrier are bent in opposite directions, and the energy levels of adjacent quantum wells are different. When a forward bias is applied, the electric field in the InGaN barrier (QB) is weakened because of the cancelation of the initial polarization electric field by the applied electric field, so that the energy difference between adjacent quantum wells is reduced and the coupling is enhanced as seen in Figure 1b. The external field tunes the position of the ground state of each QW layer by changing the band bending of the well and barrier layer. In other words, an electric field can be used to adjust the coupling degree of adjacent QWs and then tune their coupling state. However, when the applied forward bias is further increased, the tilt of the QB energy bands may be strengthened in the opposite direction, and the energy difference may be increased, which means the coupling becomes weakened again, as seen in Figure 1c. So, the “modest forward bias” in Figure 1b means that the value of the electric field intensity in the active region induced by the applied bias must be suitable.

Similar coupled state transitions may be observed in PL as EL. This is because the Coulomb shielding of the carriers in the active region in PL can also be used to shield the polarization field [12]. The photon-generated carriers diffuse from QW to the n-GaN and p-GaN layers, respectively, and accumulate. Similar to what happens in the photovoltaic effect, an open circuit voltage (V_oc_) is formed, which is opposite to the built-in electric field (V_int_) of the p-n junction [15]. Kim et al. [16] measured the V_oc_ value and found it comparable to Vint at a given optical pumping intensity. The effect of V_oc_ in PL is similar to the applied forward bias in EL. V_oc_ modifies the band structure of the active region from the structure shown in Figure 1a,b by increasing the excitation power, meaning that the active region is switching from the “uncoupled” to the “coupled” state. This explains why the CQW structure switches from unimodal luminescence at low power density, to bimodal luminescence as the excitation power density increases.

In this paper, luminescence properties of InGaN/GaN QW with a 3/5 nm well/barrier width are investigated by room temperature (RT) PL and EL spectroscopy. The effect of the electric field on the coupling between the InGaN/GaN QW is explored by comparing and analyzing the behavior of the peak energy, full width at half maximum(FWHM), and integrated intensity of PL and EL spectra. Our results have implications for the understanding of the basic luminescence properties of InGaN/GaN CQWs and pave the way for the effective fabrication of optoelectronic devices based on these structures.

## 2. Materials and Methods

**Figure 2:**
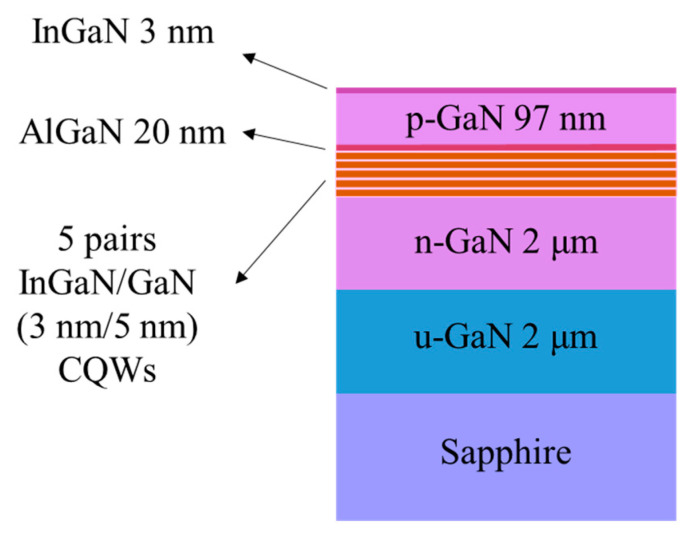
Schematic cross-sectional diagram of the violet InGaN/GaN coupled QW structure.

Figure 2 shows a schematic cross-sectional diagram of the violet InGaN/GaN CQW structure. The device is grown on a c-plane sapphire substrate via metalorganic chemical vapor deposition (MOCVD). The active layer consists of 5 periods of InGaN/GaN (3/5 nm) QWs with a fraction of 10% of indium. PL and EL techniques are used to characterize the structure. PL is performed using a pulse laser which operates at 320 nm (5 ns, 20 Hz) as excitation sources. EL spectra are measured using a semiconductor parametric analyzer (Keithley 2400, Cleveland, OH, USA)) at different operating current densities. In both PL and EL measurements, a Princeton ACTON Spectrapro-3000i monochromator (Princeton Instruments, Trenton, NJ, USA) and a thermoelectrical-cooled Synapse CCD detector (Princeton Instruments, Trenton, NJ, USA) are used to scatter and detect the luminescence signals. The optical path is held constant to ensure identical test conditions. To fabricate LED devices, an annealed 60 nm thick indium tin oxide (ITO) film serves as the p-type Ohmic contact layer. Subsequently, an area of 300 μm × 300 μm is selected by etching down n-type GaN by inductively coupled plasma etching (ICP). Finally, Cr/Au (30 nm/150 nm) multilayers are deposited on both p-type ITO and n-type n-GaN electrodes.

## 3. Results and Discussion

**Figure 3:**
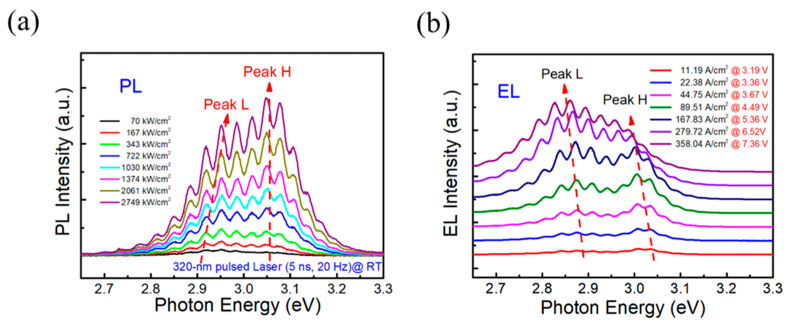
Excitation-power-dependent (**a**) PL spectra from 70 kW/cm^2^ to 2749 kW/cm^2^ and (**b**) EL spectra at various injection currents density.

**Figure 4:**
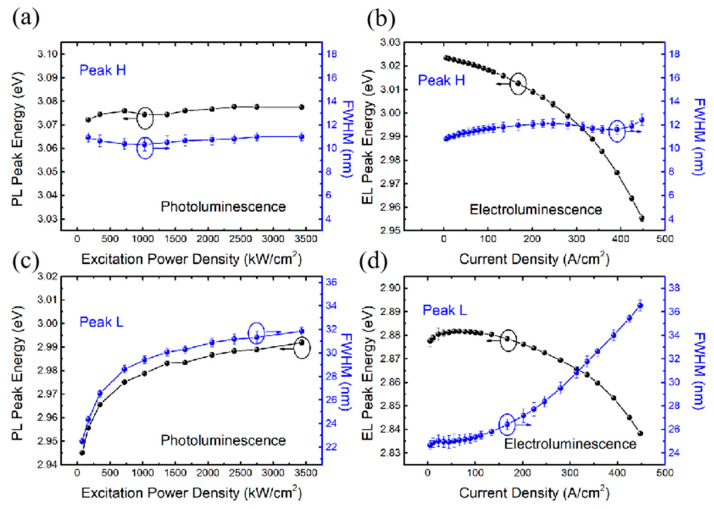
The peak energy and the corresponding FWHM of (**a**) PL and (**b**) EL of Peak H as functions of the excitation power and current density, respectively. The peak energy and FWHM of (**c**) PL and (**d**) EL of Peak L as functions of excitation power and current density, respectively.

Figure 3 shows PL (a) and EL (b) spectra at different excitation powers at room temperature (RT), respectively. As is apparent from the plots, bimodal luminescence was observed in both PL and EL spectra. Assuming that the QWs are identical, the appearance of bimodal luminescence is associated with the adjacent wells’ coupling resonance. As a consequence, the ground state energy level of the QW splits from a single state into a symmetric and an antisymmetric one relative to the barrier center [13,14], thus leading to multi-level luminescence. In the following, we refer to the peak with higher energy as Peak H and the other one as Peak L. Peak H comes from the energy of the AS-AS level transition while Peak L comes from the SS-SS state (see Figure 1b). The optical behaviors observed in PL and EL spectra are clearly different. In order to further study the luminescence characteristics of CQWs in PL and EL, the peak energy and the corresponding FWHM were analyzed using Gaussian fitting, and the results are shown in Figure 4. The interference peaks are observed in the PL and EL spectra because of the interference effect between air/GaN and GaN/sapphire interfaces that formed a Fabry–Perot microcavity [17].

In PL measurements, the peak energy and FWHM of Peak H remain nearly constant with increasing excitation power density (see Figure 4a), whereas for Peak L, the peak energy exhibits gradual blueshifts (~45 meV) and the FWHM keeps widening (from ~22 nm to ~32 nm, see Figure 4c). This behavior well reflects the In-cluster effect in the InGaN system, which leads to localized states [18]. In PL, the excitation-induced carriers will relax rapidly to the lowest energy states, that is, the localized states of the low Indium component emit light first, which results in the FWHM of Peak L being much wider than Peak H. Meanwhile, the band-filling effect in the localized states plays a leading role in the low excitation power density range (70~1030 kW/cm^2^), which is consistent with the characteristics of Peak L (rapidly blue shift and FWHM broadening). With further raising the power density, the localized states will be saturated then a slow variation trend appears in the peak energy and FWHM for Peak L.

In EL measurements, the energy of Peak H is redshifted with increasing current density [see Figure 4b] whereas the FWHM is nearly constant. The energy is redshifted also for Peak L, whereas the FWHM markedly increases (as it also happens in PL measurements). In other words, the FWHM of Peak L is broadening with the increase in carrier density in both cases (PL and EL) though at different speeds (see Figure 4c,d). For a better comparison, one may estimate the carrier density generated in the active region by using the injection power density calculation formula [19]:

(1) $$Injected\;Carrier\;Density=\frac{P}{f\times \left(h\nu \right)\times d_{active}}\times {exp}^{\left(-\alpha_{GaN}d_{GaN}\right)}\times {[1-exp}^{\left(-\alpha_{InGaN}d_{active}\right)}]\times (1-R)$$

where P, f, and hv are the power density, repetition rate of the excitation sources, and the energy of the injected photon (hv), respectively; α_GaN_ and α_InGaN_ are the absorption coefficients of GaN and InGaN at excitation wavelengths as obtained from the literature [20]; d_active_ and d_GaN_ are the thicknesses of active region and GaN, respectively; and R is the reflectance at the excitation wavelength of GaN surface, calculated as follows [21]:

(2) $$R=\frac{n_{GaN}-n_{air}}{n_{GaN}+n_{air}}$$

where n_GaN_ = 2.50 and n_air_ = 1 are the refractive index of GaN and air at the excitation wavelength. The corresponding injected carrier density ranges from 10^16^ cm^−3^ to 10^17^ cm^−3^. The concentration of carriers in the active region of 300 μm * 300 μm LED at 100 A/cm^2^ is of the order of 10^18^ cm^−3^ [22]. In these high carrier injection conditions, localized states tend to gradually saturate, which in turn leads to the observed difference between PL and EL results. As may be seen from Figure 4d, EL FWHM broadening occurs under large current density, due to non-negligible thermal effects.

An important factor affecting the features of luminescence spectra is the electric field since it will affect the coupling of QWs. In fact, the V_oc_ generated during PL measurements has a small variation range [23], smaller than the applied voltage in the EL test. Particularly, the applied voltage during EL measurements ranges from 3.2 V to ~7.4 V, which covers a large voltage range. Figure 1b,c show the influence of different voltages on the energy band of the active layer in EL. When a medium voltage is applied, the ground state energy level difference between the left and right wells decreases, and the coupling is strong. The energy level difference between the AS and SS is large, as shown in Figure 1b. When the applied voltage is further increased, the polarization of the well layer increases as well, while that of the barrier layer is lowered. This increases the difference between the ground state levels of the left and the right wells, which in turn decreases the coupling. As a result, the energy of the AS-AS level transition (Peak H) decreases while that of the SS-SS state increases (Peak L), for the AS and SS are both approaching the uncoupled state. At a higher voltage, the QWs may uncouple, as shown in Figure 1c. This is also consistent with the results shown in Figure 4b,d, where the energy of Peak H decreases with the increase in current density (increase in applied voltage), while that of Peak L increases within the range 10~30 A/cm^2^.

**Figure 5:**
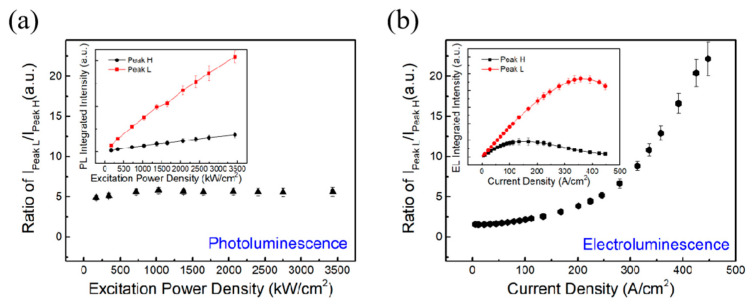
The ratio between integrated intensities of Peak L and Peak H in (**a**) PL and (**b**) EL. The inset shows the dependence of the integrated intensities of Peak L and Peak H on the excitation power and current density, respectively. I_Peak L_ and I_Peak H_ denote the integrated intensities of Peak L and Peak H, respectively.

The integrated intensities of the two peaks were analyzed by Gaussian fitting. Figure 5a,b show the ratio between the integrated intensities of Peak L and Peak H in PL and EL as a function of the excitation power and current density, respectively. In PL, with the increase in excitation power density, the ratio of I_Peak L_/I_Peak H_ remains almost constant at around ~5. In EL, the ratio increases with the injection current density, indicating that Peak L contributes a larger fraction of the whole luminescence spectrum. The inset shows the dependence of the integrated intensities on the excitation power and current density, respectively. In the inset of Figure 5a, it can be seen that Peak H and Peak L both increase linearly and there is no saturation, whereas in the inset of Figure 5b, we see that both the integrated intensities of Peak H and Peak L decrease at large current density. The decrease in Peak L is related to the thermal rollover phenomenon in LED, which is due to the thermal effect in EL. However, there is a decrease in the integrated intensities for Peak H at 100 A/cm^2^, which is mainly related to the modulation of the electric field into the QW. As the applied voltage increases, the QW coupling becomes weaker and weaker, so the integrated intensity of Peak H first linearly increases and then gradually decreases. On the other hand, the spectrum of Peak L is mostly affected by the localized states, so the weight of Peak L in the whole spectrum becomes stronger and stronger.

**Figure 6:**
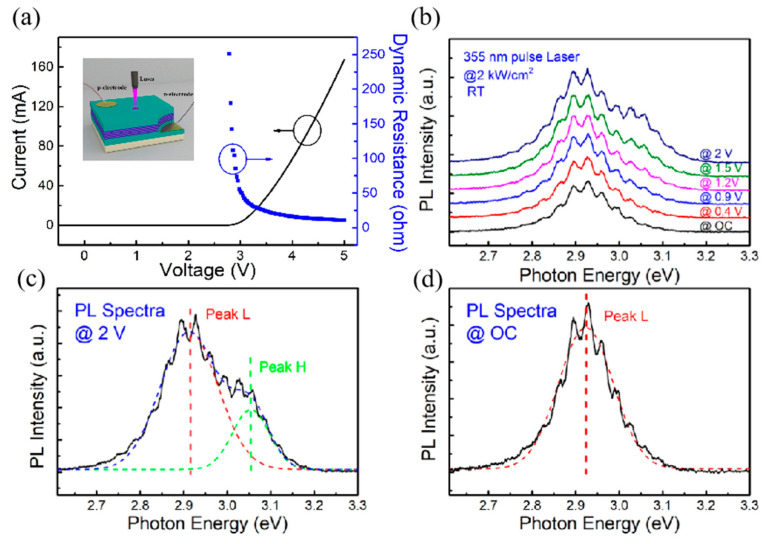
(**a**) I–V curve and dynamic resistance characteristics of the LED. The inset displays the schematic diagram of the PL experimental setup modulated by the applied voltage. (**b**) PL spectra at 2 kW/cm^2^ LED excitation power and different voltages. The PL spectra and the fitting curves are shown at (**c**) 2 V and (**d**) zero bias condition.

In order to assess the modulation effect of applied voltage, we perform further experimental measurements. Figure 6a shows the I–V curve and dynamic resistance characteristics of the LED. The inset displays the schematic diagram of the PL experimental setup modulated by the applied voltage. The turn-on voltage of this device is 2.8 V, so the applied voltage in the PL experiment is less than 2 V to make sure there is no electroluminescence in the spectra. A 355 nm pulsed laser is used as a source of lower excitation power density (2 kW/cm^2^) to obtain unimodal luminescence in the active region and the V_OC_ value is ~1 V. We send the laser beam on the upper surface of the LED and keep it irradiated then connect the electrodes on n- and p-side to a Keithley 2400 source meter. We keep the laser power density constant and modulate the voltage applied to the LED. PL results together with the corresponding Gaussian fits are shown in Figure 6b–d for 2 V and open circuit (OC), respectively. As can be seen from the figures, for OC, the PL spectrum of LED shows unimodal luminescence. When a forward bias is applied to the LED, the PL spectrum gradually becomes bimodal, which is consistent with the analysis reported above. These results provide useful guidance for the design and fabrication of optoelectronic devices, especially devices with a resonant cavity. In microcavity optoelectronic devices, the coupling between a cavity mode and material gain plays an important role. The modulation effect of applied voltage on PL spectra means that the material gain will also shift with the applied voltage. The shift of the gain peak will degrade the coupling status and then the performance of the devices. Given that the good coupling between electrons and photons at resonant modes is desirable when designing and fabricating microcavity-based devices, the modulation effect of applied voltage is an important factor.

## 4. Conclusions

In summary, the optical properties of purple InGaN/GaN CQW structures have been studied by PL and EL. Due to the coupling effect of InGaN QWs, high energy emission peaks and low energy emission peaks (Peak H and Peak L) related to the splitting of ground state energy levels have been observed. Due to the difference in the electric field direction and net polarization direction, the energy level of QW can be changed by modulating the tilt of the energy band of the well and barrier layer. This is obtained by applying an external electric field as carried out in EL measurement. Therefore, different optical properties were observed for PL and EL. Our results are useful for the study of the physical properties of InGaN/GaN CQW structures and for the development of novel optoelectronic devices.

## Data Availability

The original contributions presented in the study are included in the article, further inquiries can be directed to the corresponding authors.
